# Sharing the spotlight: Uncovering common attentional dynamics across species

**DOI:** 10.1371/journal.pcbi.1014191

**Published:** 2026-04-22

**Authors:** Mina Glukhova, Alejandro Tlaie, Robert Taylor, Pierre-Antoine Ferracci, Katharine Shapcott, Berkutay Mert, Olga Arne, Andrei Ciuparu, Raul C. Muresan, Martha N. Havenith, Marieke L. Schölvinck

**Affiliations:** 1 Ernst Strüngmann Institute for Neuroscience in cooperation with the Max Planck Society, Zero-Noise Lab, Frankfurt am Main, Germany; 2 Goethe University Frankfurt, Faculty of Biological Sciences, Frankfurt am Main, Germany; 3 Transylvanian Institute of Neuroscience, Experimental and Theoretical Neuroscience Lab, Cluj-Napoca, Romania; 4 STAR-UBB Institute, Babeș-Bolyai University, Cluj-Napoca, Romania; Utrecht University: Universiteit Utrecht, NETHERLANDS, KINGDOM OF THE

## Abstract

Sustained attention is a key underlying process to many natural behaviours that are shared across species. Yet the way attention is commonly studied in a lab context precludes meaningful cross-species comparisons. Here, we engaged mice, monkeys, and humans in the same, naturalistic perceptual decision task in a virtual reality environment. We captured their behaviour in several parameters along the speed/accuracy axes along which sustained attention is classically defined, and used Hidden Markov Models (HMMs) to infer four attentional states. We show that the dynamics of these states, both in terms of their durations and transitions, are more similar across species than might have been expected. Moreover, attentional state fluctuations seem to be internally generated and are not predicted by task attributes. The task and analyses developed here represent a new approach to infer the dynamics of sustained attention from naturalistic behaviours, in a way that is generalizable across species.

## Author summary

All animals need attention to survive - it helps to focus on what is important and ignore distractions, sometimes for extended periods. But attention is not steady; it fluctuates naturally. To understand how attention works, it is useful to compare how it functions across different species. Most studies, however, look at attention in simplistic and artificial settings, making this comparison difficult. In this study, we engaged mice, monkeys, and humans in the same, naturalistic task within a virtual reality environment. By tracking behaviour over time, we identified distinct attention states and how they change during task performance. We found that all three species showed surprisingly similar patterns of attentional fluctuations, suggesting that the mechanisms behind this process may be conserved. Our work shows that using more intuitive and naturalistic tasks can help understand fundamental cognitive processes and provide insights into attention-related disorders such as ADHD.

## Introduction

Compared to insects and invertebrates, many mammals have a large repertoire of flexible behaviours, such as foraging, scanning the environment for potential dangers, and social interactions. For many of these behaviours, attention is a key modulatory cognitive process. Without sustained attention, mice would be easily caught by predator birds; monkeys would not find enough food; and humans would be unable to read scientific papers.

This type of sustained attention towards one’s environment and behaviour has been captured in a variety of partially overlapping yet distinct terms, including task engagement, vigilance, and tonic alertness, to name but a few. Despite their differences, the central shared hallmark of all these constructs is clear: better task performance. When animals or humans attend to a task, they typically show faster and less variable response times [1,2] and more consistently correct responses [3,4]. A state of high sustained attention has also been shown to manifest itself in certain physiological parameters, such as increased pupil size [5,6] and locomotion [7].

In a scientific context, sustained attention is mostly operationalised by continuous performance tasks (CPTs); repetitive tasks that require participants to maintain focus in order to respond to infrequently presented targets or inhibit responses to foils. In a standard visual CPT for humans, letters flash on a screen, and the participant must quickly press a button whenever an infrequent and unpredictable target letter appears [8]. Performance is gauged by the accuracy of the responses, captured in the number of hits, misses, and false alarms (i.e., erroneous responses to non-targets); as well as by response speed (i.e., reaction times).

To study sustained attention in animals, analogue CPTs have been developed. In the 5-Choice Serial Reaction Time Task (5-CSRTT), a rodent is required to continuously monitor five locations in a row for a brief light flash in one of them, after which it must quickly nose-poke the location where the flash appeared to receive a food reward [9–11]. Key performance measures are percent accuracy, omission rate, and preemptive reaction times (to gauge impulsivity). To better align the 5-CSRTT with human CPTs, recent adaptations include trials on which no flash is presented, thus opening up the possibility for false alarms as well [12]. In contrast, CPT tasks in primates resemble human CPTs more, requiring the monkeys to respond to repetitive, abstract stimuli (not letters) on a computer screen [13,14].

The CPT in its various forms has led to a broad convergence in how sustained attention is defined in human and animal research. In both domains, the core idea is that sustained attention is an attentional capacity that enables stable, goal-directed performance over time. Behavioural markers like hits, misses, false alarms, and reaction speed are the most common instrumentalisations across species. Moreover, in different species, sustained attention can be taxed by either increasing task demands or the total time over which attention has to be maintained [15,16]; and pharmacological manipulations known to affect human CPT performance produce analogous effects in animals [17], reinforcing the notion that the construct of sustained attention, as measured by CPTs, has general cross-species validity at a behavioural and neural level.

Despite this, CPTs face two serious problems. First, the inactive nature of the task, which requires the subject to only sustain their attention for infrequent targets while abstaining from engaging in any other activity, fails to emulate circumstances under which sustained attention would naturally be employed by different species. For instance, foraging behaviour relies on sustained attention, with attention modifying the performance of the animal on the underlying cognitive process, in this case perceptual decision-making. As such, CPTs by definition require unnatural and restrictive behaviours, which may explain the sometimes considerable training durations they require, e.g., for rodents to reach strong task performance. This makes it difficult to determine to what extent findings based on classical CPTs generalise to other task paradigms - and even more importantly, how well they reflect attentional fluctuations that might occur spontaneously in the wild. Second, the implementation of CPTs does not translate well across species. For instance, human CPTs rely on instructions and can incorporate complex rules or stimuli, whereas animal tasks must be simplified to easily perceivable cues and require extensive training with reward feedback. More importantly, rodent CPTs involve strong motor components, such as repeatedly traversing an experimental chamber to reach different nose poke locations, that are completely absent from human and primate CPTs. Lastly, humans typically produce variable reaction time data, whereas rodents and primates, due to extensive overtraining, tend to respond as soon as they detect a stimulus. Consequently, human studies often emphasise reaction time variability as an informative measure of attentional fluctuations, whereas animal studies emphasise accuracy and omissions as the clearest readouts of attention [17].

Taken together, these vastly different implementations of the CPT prohibit meaningful cross-species comparisons of sustained attention as a principal and innate cognitive process. Given that attention is such a fundamental building block of cognition, it seems reasonable to assume that dynamically fluctuating attention is a process that is largely shared across species, but current paradigms have faced difficulties observing this process ‘in its natural habitat’.

In order to study sustained attention in natural behaviour across species, we engaged mice, monkeys, and humans in the exact same dynamic perceptual decision-making task. To facilitate a comparison of spontaneous attentional dynamics across species, we made the task as naturalistic as possible: Instead of an abstract mapping of, e.g., button presses to artificial stimuli, stimuli resembling natural objects were displayed in an immersive virtual reality (VR) environment displaying a photo-realistic natural scene. Hence, the visual input was more dynamic and varied than in a typical decision-making task, including visual flow, variable viewing angles of the objects initiated by their independent movement in conjunction with the subject’s own movement, and natural (and hence irregular) visual textures across the scene. Subjects of all three species were required to navigate towards targets (for mice by running on a spherical treadmill, for monkeys and humans by moving a trackball with their hands). To encourage a feeling of natural hunting, the targets were moving through the VR environment at the start of each trial. To further ensure naturalistic attentional processing of stimuli, subjects could freely move their eyes.

Due to these features, our VR decision-making task is more intuitive to perform for all three species. What’s more, this approach also allows us to capture behaviour in a much richer and more nuanced way than classical experimental paradigms. By analysing the trajectories that subjects take through the VR on each trial, we quantify their behaviour in a host of parameters beyond the classical reaction time (RT) and task accuracy (correct/incorrect). This enables the quantification of performance strategies in single trials. For example, a high RT on a particular trial might indicate that an animal is strongly engaged in the task but unsure of the right answer (high level of attention), or that its focus is drifting off (low level of attention). Such differences can manifest in behavioural parameters such as the precision with which stimuli are approached in the VR environment. As such, these parameters together give a more accurate manifestation of the attentional state of the subject.

With this approach, we for the first time directly compare the dynamics of spontaneous attentional fluctuations across three species and on a trial-by-trial basis, as well as the strategies they use to allocate fluctuating attentional resources to increase either response speed, accuracy, or both. Our setting is both naturalistic, replicable across species, and allows tracking attentional fluctuations on a single-trial basis based on a rich arsenal of behavioural readouts.

## Results

### Experimental design

**Fig 1:**
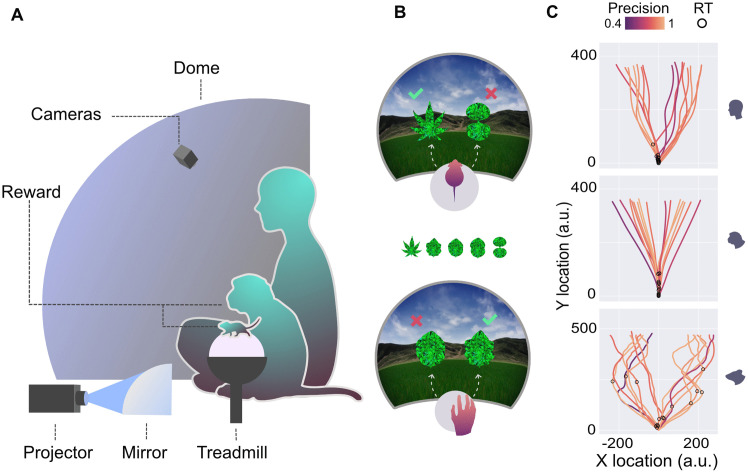
Experimental setup and path parameters. A) Mice, monkeys, and humans were placed inside a large dome, on which the VR task was projected via a curved mirror. Mice traversed the VR by running on a trackball, while monkeys and humans did so by moving a trackball with their hands. B) The perceptual decision task involved moving towards either of two stimuli, a rewarded target (check mark) or an unrewarded distractor (x). Targets and distractors were selected from a range of morphed shapes between a pointy leaf and an hourglass (see Methods). C) The paths of the humans (top), monkeys (middle), and mice (bottom) towards the stimulus on several trials. Open circles denote reaction times and path colour denotes the ‘precision’ metric (see Methods - Metrics for the definition).

To compare their attentional fluctuations, we engaged 3 mice, 2 monkeys, and 11 humans in a simple two-alternative non-forced choice task, where they had to discriminate two leaf-shaped objects (Fig 1B). To elicit naturalistic fluctuations in attention, we employed an intuitive task structure (navigating towards food sources) based on naturalistic visual stimuli: leaves placed in a virtual reality (VR) environment consisting of a realistic grassy field and mountainous background, projected into a highly immersive dome surrounding the subjects (Fig 1A; see also [18]). The mice traversed this VR environment towards either object by running on a styrofoam ball; the monkeys and humans used a large trackball for this that they could operate with either one or both hands (monkeys), or the right hand only (humans). Subjects were freely viewing the stimuli and the environment.

We used the subjects’ trajectories through the virtual environment to capture their task performance in five parameters, mapped onto the two seminal axes of attention: accuracy and speed. Capturing each axis by more than one behavioural parameter allowed us to make more nuanced estimates of speed and accuracy on a single-trial basis, beyond what can be achieved, e.g., by binary classifications of hit and miss trials.

Three parameters were used to gauge task accuracy: *instantaneous hit rate* (correct vs incorrect or miss trials; henceforth indicated as hit rate), *precision* (how accurately subjects hit either of the stimuli relative to average successful hit location; see Fig 1C), and *bias* (the tendency to repeatedly approach objects on the same side, regardless of their identity). Both changes in precision and bias have previously been associated with different attentional states in mice [4,19]. The remaining two parameters specified speed: *reaction time* (the point in time the subject makes a turn towards one of the objects; see Fig 1C), and *speed* (average speed of moving through the VR environment from trial start to end). (For exact parameter definitions, see Methods - Metrics; for the distributions of these parameters for the three species, see S1 Fig.)

### Models

**Fig 2:**
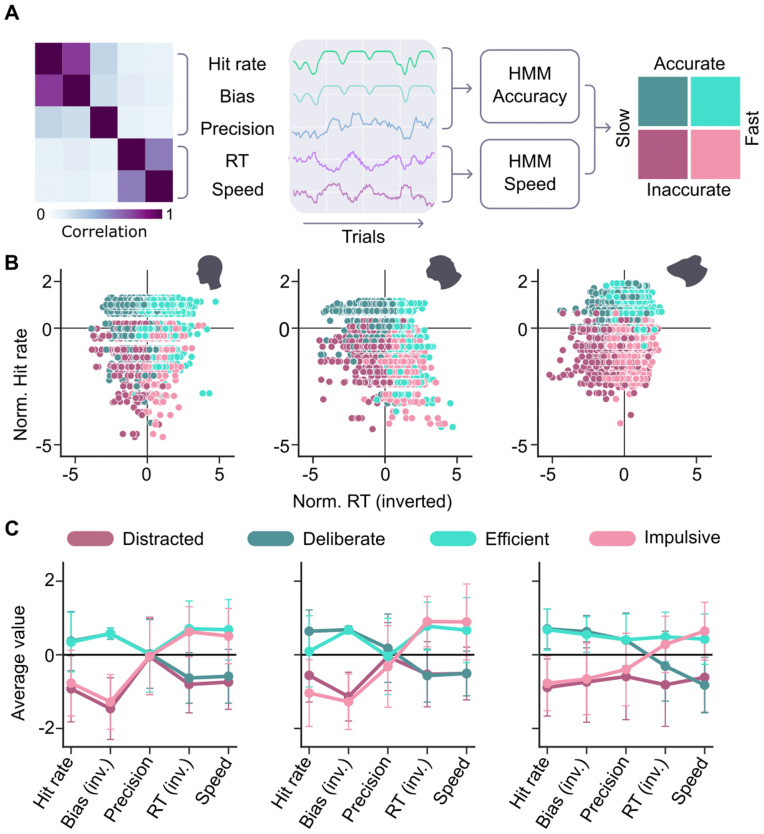
Attentional metrics and models. A) Five variables (middle) were derived from the VR paths: three concerning task accuracy (hit rate, precision, and bias) and two concerning speed (running speed and reaction time, RT). The correlation matrix of these variables (left) confirmed that the variables concerning the classes accuracy and speed were highly correlated within each class, but largely independent between the two classes, though precision showed only a weak association with either. The variables were therefore entered into two separate HMMs, the conjunction of which partitioned the data into four categories (right). B) Plotting a representative variable from each of the two classes (hit rate and reaction time) against each other and colouring the data by the four states, confirmed a correct partitioning of the data by the HMM conjunction, for humans (left), monkeys (middle), and mice (right). Legend of state colours: see C. C) The contribution of the five variables towards the partitioning into four states by the conjuncted HMMs was very similar between humans (left) and monkeys (middle). In mice (right), the precision variable had slightly different contributions towards the four states, but the overall pattern was similar to that of humans and monkeys. For this analysis, reaction time, bias and precision were inverted (marked as inv. in the labels) so that higher values in all metrics represent higher performance. All metrics were normalised to each species’ average values.

The choice to categorise parameters as contributing either to the speed or accuracy dimension of task performance was made for conceptual reasons: we wanted to know how subjects navigated the well-known trade-off between speed and accuracy given their available attentional resources. This trade-off has classically been considered a fundamental feature of attentional processing [20]. To verify to what extent this conceptually driven choice tallied with the actual structure of our behavioural data, we quantified the relations between the five behavioural parameters (Fig 2A, middle). Specifically, we correlated the parameters’ overall time series (i.e., one value per trial, with all sessions within one species concatenated). The resulting correlation matrix consistently showed two clusters of correlated parameters: ‘hit rate’ and ‘bias’ as metrics quantifying performance accuracy, and ‘reaction time’, and ‘running speed’ as metrics quantifying performance speed, with low correlations between the two clusters (Fig 2A, left). Hierarchical clustering with silhouette analysis confirmed this two-cluster structure across all three species (S2 Fig). This pattern is in line with the notion that behavioural parameters cluster onto the two axes of accuracy and speed, along which task performance can fluctuate independently.

Note however that the ‘precision’ variable did not seem to correlate strongly with either cluster, as reflected by its consistently low silhouette scores (0.05 - 0.17 across species, S2 Fig). Based on our conceptual rationale for including parameters in the two HMMs, we chose to include ‘precision’ in the ‘accuracy’ cluster - both because conceptually it captures aspects of performance accuracy rather than speed, and because empirically, ‘precision’ generally showed somewhat closer clustering with ‘hit rate’ and ‘bias’ than with ‘reaction time’, and ‘running speed’.

Attention is not the only factor shaping performance speed and accuracy - external factors like stimulus difficulty and history on a particular trial also play a decisive role. We hypothesised that dynamic changes in the two parameter clusters should reflect fluctuations in sustained attention beyond the performance differences elicited by specific trial attributes. To better capture such spontaneous fluctuations and minimise the contribution of trial attributes, we first smoothed the time series with a window size of 5 trials to reduce the influence of individual trial factors like stimulus difficulty (see Methods, also S3 Fig). To parcellate the time series into distinct attentional states, we used two independent Hidden Markov Models (HMMs; for details on the models, see Methods - HMMs). The accuracy and speed parameters were used as inputs into the two separate HMMs, each outputting two states: one model differentiating between slow and fast states, and one model differentiating between accurate and inaccurate states. The two HMMs were fit separately for each species; so even though humans and monkeys have generally better task performance than mice in absolute terms, we defined high- and low-performance states based on relative task performance per species. However, fitting the two HMMs on data of all species combined resulted in very similar state profiles (S4 Fig). The conjunction of the two HMM outputs resulted in four distinct state classifications: *distracted* (low accuracy and low speed), *deliberate* (high accuracy and low speed), *impulsive* (low accuracy and high speed), and *efficient* (high accuracy and high speed) (Fig 2A, right). Each task trial was labelled with one of these four states. It should be noted that these labels are intended solely for readability and are not meant to carry the connotations typically associated with terms like impulsivity or deliberateness in the literature [21,22].

To verify that these four HMM-derived states did not represent random data partitions, but could be related to more classical behavioural metrics of speed and accuracy, we visualised trials in the space of hit rate versus reaction time, coloured by their assigned state (Fig 2B). We observed that our states generally fall within the expected quadrants of the space (e.g., ‘efficient’ trials showing both fast reactions and high accuracy). In contrast, fitting a single 4-state HMM to all five behavioural variables yielded state profiles that were difficult to interpret, with inconsistent contributions of the different parameters (S5 Fig). However, some trials fall in other quadrants than one might expect based on a simple cut-off criterion. This is the case because by design the HMMs did not simply classify states linearly, but also captured temporal dependencies between trials by formalising them as state-to-state transition probabilities learned from the data. The HMM-generated states also appeared to partition trials sensibly for other read-out parameters like bias and running speed. One clear exception was the ‘precision’ parameter in monkeys and humans, which was not well-partitioned by the HMM states. This is consistent with the fact that precision is only weakly correlated with our speed and accuracy metrics, which suggests that it may capture a partially distinct aspect of performance, potentially more related to motor execution than to the perceptual decision-making. Another exception can be seen for reaction times in mice. This might be due to the mice continuously moving on the treadmill, potentially making reaction times more dependent on movement context (e.g., running speed at trial start) and more difficult to determine precisely (S6 Fig). Taken together, these observations support the use of the two-HMM architecture as a simple, hypothesis-driven way to characterise attentional fluctuations along speed and accuracy dimensions across species.

To complement the partitioning analysis, we investigated the relative representations of all five parameters in the two HMMs. As expected, in the accurate states, the three accuracy parameters had higher average values (and vice versa for the inaccurate states), whereas in the fast states, the speed parameters had higher means (Fig 2C). Importantly, this pattern appeared similar across the three species, indicating that these input parameters, despite being recorded in different ways (actual running for the mice, moving a trackball with the hand for monkeys and humans), all contributed similarly to defining each attentional state. Statistical tests of cross-species pattern similarity (see Methods, Analysis of dwell times and transitions) confirmed this finding (human-monkey: r = 0.96; human-mouse: r = 0.88; monkey-mouse: r = 0.87), even though the Euclidean distance between the parameter distributions of the different species was larger than expected by chance (p ≤ 0.015). Thus, the overall pattern of metric combinations that define each state appears conserved across species, but with significant differences in the exact values.

### State characterization

**Fig 3:**
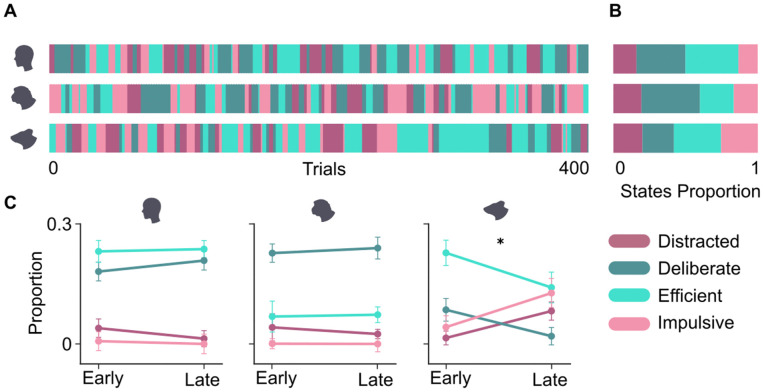
Model comparisons across species. A) Partitioning the trials of an example session of humans (top), monkeys (middle) and mice (bottom) into the four states showed both long (several tens of trials) and short (just a few trials) stretches of a particular state. B) Overall time spent in each state was very similar for humans and monkeys, whereas mice spent less time in the deliberate, and more time in the impulsive state. C) Proportion of time spent in each attentional state during the first and second half of sessions, shown separately for humans, monkeys, and mice. The asterisk denotes significant differences for all states (p < 0.05, permutation test).

Visualising states in time showed participants dwelling in states for several trials before transitioning to the next state (see Fig 3A for an example session). Across species, participants spent relatively more time in the accurate states (deliberate and efficient) than in the inaccurate states (distracted and impulsive) (Fig 3B). Statistical tests (see Methods, State occupancy across species) showed that the proportion of the distracted state was indeed not significantly different between the species (p = 0.22), whereas the proportions of the remaining three states showed significant differences: mice spent comparatively less time in the deliberate (p = 0.0002) and more time in the impulsive (p = 0.003) state, and monkeys spent less time in the efficient state (p = 0.002) than the other two species. Note that in the ‘distracted’ state, not all trials necessarily had slow and incorrect outcomes; just comparatively more than during the efficient (fast and correct) state.

One potential confound is that performance speed and accuracy may be strongly shaped by internal variables that are not directly related to fluctuations in sustained attention, for instance, slow changes in fatigue (or in the case of monkeys and mice, satiety) throughout a session. While the drift correction implemented on the behavioural variables used for our HMM analysis (see Methods, Preprocessing) should account for such slow changes to some extent, they may still bias our analysis throughout the time course of a session. To check whether slow drifts in internal variables were driving the occurrence of our four performance states, we computed the relative proportion of the four states over the course of each session by splitting sessions into an early and a late half. As shown in Fig 3C, the proportions of the four different states remained stable between the first and second half of the session for humans and monkeys (permutation tests yielded p-values > 0.05, see Methods). In contrast, mice became progressively less accurate over time (p < 0.05, see Methods), possibly reflecting increasing fatigue or satiation. This suggests that the state transitions inferred here were not mainly driven by slow internal drifts, and can thus be seen as a viable proxy for more dynamic (presumably attention-driven) fluctuations.

### State dynamics

**Fig 4:**
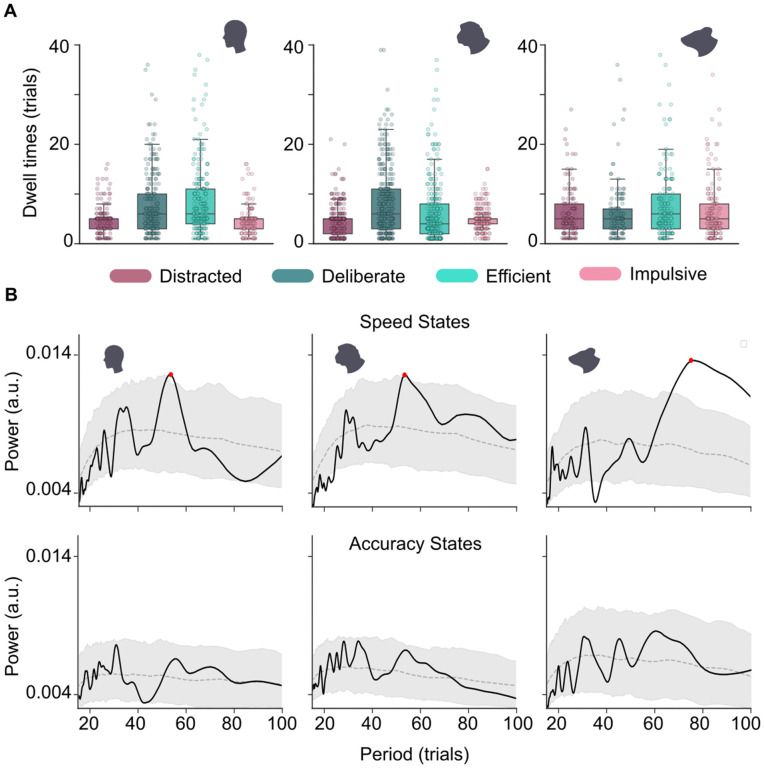
State temporal dynamics. A) Dwell times show a large spread, but are on average very similar between the species. Within each species, interesting differences emerged; for instance, humans, tended to stay longer within an accurate than within an inaccurate state. B) Frequency analysis on the speed states (top) of humans (left), monkeys (middle) and mice (right) revealed a similar frequency peak in humans and monkeys around 55 trials, and a peak around 75 trials in mice. The grey area denotes the 95 percent confidence interval of shuffled trials (see Methods -Shuffling). A red mark indicates a peak exceeding the 95% shuffled confidence interval. Bottom: same, but then for accuracy states.

Beyond the overall proportions at which the four different states occurred across species (see Fig 3B), their distribution over time may also vary. Anecdotally, many neuroscientists are familiar with the hugely different attention spans of different species; mice can often only be trained for 20–30 minutes at a time, whereas a typical human experiment lasts about an hour, and for a well-trained monkey, training times of up to 2–3 hours are not unusual. This suggests that state durations in mice might be shorter, and state transitions might occur more often than in humans and monkeys. To test this hypothesis, we computed the dwell times for all states (Fig 4A). This showed that durations spent in each of the four states were short, with a median of 5 trials and an interquartile range of roughly 3–8 trials across all species. Overall, species differences in dwell time were statistically significant but numerically small (mean dwell times between 6.2 and 6.9 trials; Kruskal-Wallis test, H = 9.68, p = 0.008; see Methods, Analysis of dwell times and transitions). For example, humans and monkeys spent comparatively longer uninterrupted sequences of trials in accurate than in inaccurate states (Fig 4A, left and middle); this was not the case for mice (Fig 4A, right). Control analyses with smoothing over window sizes between 2 and 20 trials confirmed that dwell times scale linearly with window size, for all states and in all species; therefore, any differences, e.g., across species or across different attentional states, cannot have been artificially introduced by data smoothing, which was applied equally to all data (S3 Fig).

Even though the dwell times show a large spread for each species and state (Fig 4A), it could be that there is a preferred dwell time, which would imply the existence of a certain rhythm in the state durations. To examine this, we determined the frequency spectrum of the time series of all trials in all sessions (Fig 4B). As the two state sequences were largely independent (S7 Fig), we computed separate frequency spectra for each binary axis. Frequency spectra were computed using superlet analysis ([23]; see also Methods - Frequency Analysis). To our surprise, the frequency spectra along the speed axis revealed a prominent peak for all three species (Fig 4B, top). This peak was at a period of about 55 trials in humans and monkeys; for mice, it was slower, at a duration of about 75 trials (frequency spectra in the unit of seconds for all three species are shown in S8 Fig). Using session-wise peak positions within this low-frequency range (40–80 trials), we found no significant species differences in peak period (all Bonferroni-corrected p > 0.12; see Methods, Frequency Analysis). This suggests that there is an intrinsic, and potentially evolutionarily preserved, rhythm by which fast performance can typically be maintained for a period of tens of trials. Since peak frequencies can jitter across sessions, our frequency-by-frequency comparison to the shuffled spectra provides a conservative estimate of rhythmic structure; it may underestimate effects that are consistent in a broad low-frequency band but not perfectly aligned in their exact peak period. The frequency spectra of the accuracy axis revealed peaks at similar frequencies, however, these were much smaller and did not exceed the shuffled control, suggesting that performance accuracy varied less dynamically and rhythmically than speed (Fig 4B, bottom). In both axes, several small peaks were present at a faster time scale of 20–40 trials.

To assess whether fluctuations in speed and accuracy reflect coordinated attentional dynamics or rather independent latent processes, we investigated the temporal coordination between the speed-HMM and accuracy-HMM state sequences. First, we computed transition matrices on the sequences of the four ‘composite’ states (S7 Fig). However, as the composite labels are defined as the conjunction of two separately inferred state sequences, the resulting transition matrix can be largely predicted from the transition structure of these two original sequences. This yields much higher probabilities for transitions that change only speed or only accuracy than for transitions that require both sequences to switch on the same trial (i.e., between efficient and distracted, impulsive and deliberate). We therefore compared the observed transition matrices to a null distribution generated by independently shuffling the speed and accuracy state sequences (preserving dwell-time structure) before recombining them into composite states. Indeed, compared to this null, most transition probabilities were not significantly different, with only a small number of significant deviations, mostly in monkeys (S7 Fig).

We therefore focused on whether switch events in the two sequences are temporally coupled. Using a circular-shift null that preserves each sequence’s long-term structure while disrupting its alignment to the other sequence, we found a significant dependence between speed and accuracy switching in monkeys, with a peak at a lag of one trial. In mice and humans, we did not observe any dependence. Overall, this suggests that the two sequences are switching mostly independently, with some coordination between them in monkeys (S7 Fig).

### Effect of external task attributes

**Fig 5:**
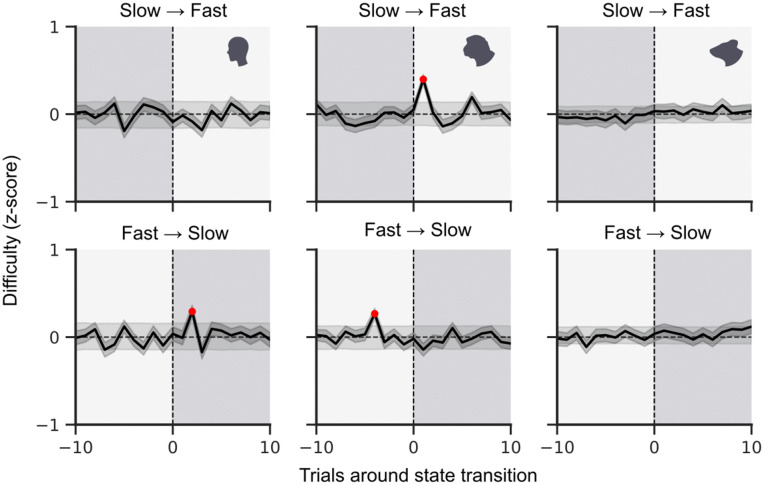
Effect of task difficulty on state transitions. Around the time of a state transition (at trial 0) from slow to fast (top row) or fast to slow (bottom row), the difficulty level of trials (black line) was not significantly different (except in three places) from the average difficulty (dotted line). The grey band shows the range of 1000 shuffled controls (see Methods). This indicates that the transitions between speed states are likely internally generated, as opposed to externally triggered, in all three species.

The presence of a rhythmic component in the speed state fluctuations (and the absence of such a rhythm in the task structure) suggests that these fluctuations are internally generated rather than task-induced. To verify this, we made use of a task attribute that strongly affects task performance, namely task difficulty [24,25]. For all three species, the task included trials of varying levels of difficulty (see Methods for details). We examined the effect of task difficulty on state transitions by computing the average difficulty of the trials around the time of each speed-state transition (slow to fast states and fast to slow states). Any effect of task difficulty would have resulted in an uneven distribution of easy and difficult trials around the time of state transition; however, this was generally not observed (Fig 5). The few significant peaks were brief and inconsistent between the species and directions; there was no sustained ramp of greater difficulty before or after transitions. In monkeys, there were brief single-trial increases of difficulty during the fast states, which is in contrast to the typical pattern, where more difficult trials lead to longer reaction times [26]. This may reflect a species-specific (or individual) strategy to quickly skip particularly difficult trials, but would only apply to a small subset of trials. This suggests that the speed state transitions are generally self-generated, even though other stimulus-related factors such as their appearance might have played a role. Repeating this analysis for the accuracy states showed a more pronounced effect of task difficulty for humans and monkeys (S9 Fig), but again restricted to a single-trial peak. This effect is expected because the model in both of those species is highly sensitive to errors. A single especially difficult trial will likely result in an error, which is more often captured by the HMM as a brief transition to the inaccurate state. A final indication that we are witnessing internally generated attentional states rather than externally induced, task-related changes in performance is given by concurrent pupil size measurements in mice and monkeys: the relationship between our five input HMM parameters and pupil size in those two species followed an inverted U-shape, such that best performance (highest hit rate, lowest reaction time, etc.) was associated with intermediate pupil size, as is commonly reported in the literature [6,27–29] (see S10 Fig).

## Discussion

Sustained attention is an important cognitive process supporting a wide range of flexible behaviours across mammalian species [30,31]. Yet the way it has been studied to date has been very species-specific, precluding meaningful cross-species comparisons. Here, we engaged mice, monkeys, and humans in the exact same, naturalistic perceptual decision task in a VR environment. We captured their behaviour in five parameters that were based on their movement trajectories through the VR, and that described their task performance either in terms of task accuracy or speed, two fundamental task-dependent variables modulated by attention [20,32]. Since our main goal was a cross-species comparison, we chose to enforce the two-axis (accuracy × speed) structure as a hypothesis-driven solution to map how different species navigate attentional states defined by the classical notion of speed-accuracy trade-offs. Speed and accuracy parameters served as inputs into two separate HMMs to infer underlying attentional states. The conjunction between these two separate HMMs partitioned the trials for all three species into four attentional states: a distracted, deliberate, efficient, and impulsive state.

We found that when attention was defined relative to the overall capacity of the animal to perform the task at hand, all three species were immersed in each of four distinct attentional states for roughly the same amount of time (Fig 3B). Moreover, they displayed the same overall behaviour; short stretches (6 trials on average, see Fig 4A) of being in the same state, with frequent transitions between them (Fig 3A). Although the short stretches of staying in the same state could vary widely in duration (Fig 4A), slow fluctuations in the balance between slow and fast states (potentially with several switches between the correct and incorrect state in between) lasted preferentially for around 55 trials for humans and monkeys, and for around 75 trials for mice (Fig 4B). In contrast, transitions between accuracy states (potentially with several switches between the slow and fast state in between) did not reveal any significant cycles. In the absence of external rhythms imposed, e.g., by shifting reward contingencies, the rhythmic transitions between fast and slow states thus suggest that these fluctuations are internally generated; the absence of an effect of task difficulty on speed state transition probability further confirms this (Fig 5). In summary, spontaneous fluctuations in attention had a similar overall structure and occurred on a comparable timescale across the three species.

HMMs, often in combination with a general linear model (GLM-HMMs) to account for the underlying states’ dependence on continuously incoming sensory information [33], have been used before to delineate states of high and low sustained attention. For example, in mice involved in perceptual decision-making tasks, a GLM-HMM trained on task accuracy distinguished between either one engaged and two disengaged states [34], or between an optimal, sub-optimal, and disengaged performance state [4]. In fact, a recent study from our laboratory trained a more recent HMM version, specifically a Markov Switching Linear Regression (MSLR) model, on the reaction times in the same visual discrimination task employed here, and revealed underlying internal states that mapped onto differences in reaction time and task accuracy in both mice and monkeys [35]. These previous studies have taken a strongly data-driven approach, allowing the number and structure of inferred states to be determined agnostically. In contrast, the present study imposed a predetermined model structure in order to explore how spontaneous behavioural dynamics map onto classical notions of attentional processing, specifically the trade-off between speed and accuracy.

It is worth noting that the attentional states described in these previous studies typically lasted much longer than the states in our data, on the order of tens up to roughly a hundred trials (c.f. example data in [34], Fig 1F and [4], Fig 1E). A possible technical explanation for this difference might be found in the fact that we enforced sets of two states onto the data, whereas the GLM-HMMs described above all had at least three states. A previous study analysing a perceptual decision-making task in mice, a two-state GLM-HMM resulted in single-trial switches, whereas a three-state GLM-HMM on the same data set yielded much slower transitions [36]. On the other hand, it is possible that our task simply induces faster state switches; the MSLR model applied in our previous work on overlapping data used three states for mice and four states for monkeys, which could technically yield longer state durations, yet showed similarly short dwell times as the current analysis [35]. One reason for this phenomenon may be that all three species operate at a high level of task performance relative to their overall ability. As a result, ‘high accuracy’ and ‘low accuracy’ states are generally quite close to each other in absolute performance. As such, they may reflect fast switches between optimal and almost-optimal performance that might not be picked up by broader (and more distinct) performance states. In this context, it is interesting to note that the emerging rhythmic switches along the speed dimension (see Fig 4) align much more closely with the state durations observed in previous studies. In other words, our analysis may be picking up on faster ‘micro-fluctuations’ that occur on top of slower rhythmic fluctuations in attentive capacity [37,38].

Overall, we found that attentional dynamics in all three species were broadly similar. Attentional state durations and transition patterns were comparable, including a clear peak on a similar timescale in the frequency spectrum. These measurements were reported in the time unit of trials; reporting the same results in seconds slightly altered these outcomes owing to the higher average trial duration for the mice (see S8 Fig as well as S11 Fig). However, trials are longer in mice largely due to a different input method for navigating the VR (which is independent from the task per se); and since all our measurements are done at trial resolution (i.e., we do not distinguish between various events within a trial), we believe that reporting our results in trials is the most accurate depiction of the attentional fluctuations.

The similarity in dynamics between the species might be expected based on the supposedly common underlying neural mechanisms of attention in primates and rodents [39,40]. Similar perceptual decision-making tasks for different species have revealed subtle differences between the species. For example, in a new version of the pulse-based evidence accumulation task (in which subjects have to select the right or left side where light pulses are flashed with the highest probability), it was shown that humans prioritise accuracy, whereas rodents favour speed of responding [41]. This is in line with our finding that mice were in a fast (efficient or impulsive) state a larger part of the time than humans and monkeys. And in an uncertain decision-making task for mice, monkeys, and humans, which was designed to investigate the balance between exploiting one strategy to solve the task and exploring alternative strategies, mice were less persistent in exploitation behaviour than primates [42]. The development of tasks such as these is crucial but difficult; even a simple component of a task, such as the same food reward across species, can have different value for these species [43].

While the task still requires training in animals, we believe it was natural enough to elicit natural fluctuations in sustained attention. The fact that the attentional fluctuations seem not to be induced by task difficulty, but rather entirely self-generated, confirms this. An experimental set-up in which the animals and humans could freely move would have allowed for natural indicators of alertness, such as ‘head scanning’ movements in rats [44], and general body and head direction in primates [45]. Even so, it is important to emphasise that VR setups (including the one we employed) are one of the only ways to keep tasks and visual input identical between different species, and are therefore ideally suited to cross-species comparisons [46]. Moreover, our VR set-up allowed us to capture behaviour in the same five parameters across the three species, which enables a direct and meaningful comparison between them.

We believe this approach has value beyond just characterising the common dynamics of sustained attention across species. A staggering 96 percent of all neuropsychiatric clinical trials fail [47]. To successfully translate results from preclinical work to the clinic, comparable and sensitive tests of cognitive processing in animals are essential [48]. However, such tests are difficult to conceive, as changes in behaviour as a result of neurological or psychiatric conditions are often difficult to reproduce in animals [49–51]. An intuitive task such as the one presented here can help refine animal models of, e.g., ADHD [52] and capture behavioural changes resulting from clinical interventions in much more detail. As such, we believe that developing tools to elicit and precisely quantify naturalistic behaviours across species in a directly comparable way is a crucial step in designing truly translatable cross-species tests of neuronal processing.

## Materials and methods

### Ethics statement

All animal experimental procedures were approved by the local ethics committee, Regierungspraesidium Darmstadt, under number F149/2000. The human experiments were approved by the ethics committee of the medical department of the Goethe University in Frankfurt under number 2021–252. Written consent was obtained from all participants prior to their participation.

### Subjects and training

3 mice (FVB/BL6, male, 10–25 weeks old), 2 monkeys (Macaca mulatta, both male, 15 years old), and 11 humans (3 male, 8 female, mean age 25 years old) were used in this study. Training sessions for mice were conducted between 10am and 1pm, and typically lasted 30–60 minutes. Note that animals were housed in a reversed day-night cycle, so that all experiments were conducted during their active phase. Mice were typically trained for 5–12 sessions until they reached a consistent performance of around 70–80% correct responses in multiple stretches throughout a session. Training sessions for monkeys were typically conducted in the morning, and lasted about 60–90 minutes. The monkeys needed about 30 training sessions before reaching near-perfect, stable performance on the perceptual decision-making task. Humans received verbal instructions and a short practice of a few minutes before conducting the experiment, typically done in the afternoon and lasting for about one hour.

### Experimental setup

Details of our experimental setup have been published previously [18]. Briefly, participants were placed inside a spherical dome (diameter 120 cm, 250°) onto which a virtual reality environment was projected via a curved mirror. The virtual reality was created using the game engine Unreal 4 and consisted of a grassy landscape with mountains in the background and a blue sky overhead, in the middle of which the two stimuli were displayed. To navigate the environment, macaques and human participants used a GK75-1602B 75mm trackball from NSI (Bilzen-Hoeselt, Belgium). Mice were placed on top of a 20 cm diameter Styrofoam ball suspended in the air (modified method from [53]). The movements of either device were translated into movement in the virtual environment.

### Experimental paradigm

All three species performed the same simple two-choice visual discrimination task. They were presented with an ambiguous blurred stimulus that immediately divided into two stimuli with naturalistic shape and texture (see Fig 1B), which then swiftly moved through the VR environment to their final position next to each other at a reasonable distance in front of the participant. One of the two stimuli, the target, resembled a pointed-lobed leaf; the other stimulus, the distractor, resembled an hourglass. The perceptual similarity between the target and distractor was varied systematically by morphing the shapes into each other. Both shapes were filled with a uniform foliage texture in a blue-green colour. The colour was selected to be easily visible both for rodents and primates. The participants were required to navigate towards and then (virtually) collide with the target while ignoring the distractor. A collision with the target resulted in a drop of soy milk for the mice, a drop of juice for the monkeys, while humans did not receive a reward. Approximately 550 ms following the collision, the next trial started. Mice and monkeys performed the task until satiated; humans performed the task until reaching a minimum of 500 trials.

The task had several difficulty levels; on easy trials, subjects had to distinguish between two shapes resembling a pointed leaf and an hourglass, whereas on difficult trials, they distinguished between two morphed stimuli only slightly resembling a pointed leaf and an hourglass. Monkeys and humans were shown shapes of comparably high difficulty. Mice were presented with more discriminable stimuli, as the goal was not to test the limits of their visual discrimination ability but to produce a reliable behavioural response.

In humans, 11 sessions were recorded, totalling 5,314 trials. Data from monkeys included 9 sessions, totalling 7,504 trials, and data from mice spanned 13 sessions, totalling 3,903 trials.

### Behavioural metrics

Each trial could have one of three outcomes: correct response (collided with the target stimulus), incorrect response (collided with the distractor) and miss (no stimulus chosen). On each trial, the path of the participant through the virtual environment was recorded and used to extract five metrics. *Instantaneous hit rate* was classified as a binary metric (correct (1) vs incorrect or miss (0)), which is later smoothed using a 5-trial sliding window during the preprocessing stage (see Methods - Data Preprocessing). *Bias* represents perseverative errors by identifying trials where the subjects repeated their previous choice despite the target appearing on the opposite side. It is a binary measure (biased (1), unbiased (0)) that reflects the tendency to choose the stimulus on the side of the previous response, regardless of the current target location. *Precision* quantifies how close each response endpoint was to the mean location of correct responses for that side (left/right), regardless of whether the current response was correct. The measure is normalised by the distance from the outer stimulus corner to the midpoint between stimuli and inverted so that higher values (0–1) indicate better precision. By computing this relative to successful responses within each session, the measure accounts for individual response biases and preferences (e.g., hitting the inner stimulus corner vs. the middle or differences between left and right responses). *Reaction Time (RT)* was defined as the first significant change in direction in the path towards one of the stimuli (see S1 Text). *Speed* refers to the average movement speed (VR units/s) of the participant in each trial.

### Data preprocessing

The data was preprocessed in several steps. First, trials with missing values were removed, as well as trials with durations over 10 seconds and extremely short travel distances (path length <250 unreal units).

In humans, there was a learning curve in motor performance that was most noticeable in the reaction time and average running speed. To remove the learning period, we calculated a combined learning cutoff based on reaction time and speed using rolling averages compared to the stable mean of the second half of each session. Trials up to the cutoff were excluded to ensure that only stabilised performance was analysed. Mice and monkeys were already proficient in the task by the time of the sessions analysed here, so all data could be used. Next, in all species we performed drift correction on the continuous metrics (reaction time, speed and precision) by fitting a second-degree polynomial and subtracting the predicted trend, to remove any extremely slow fluctuations. We subsequently smoothed the data in all species by applying a centred rolling average with a window of 5 trials to suppress noisy single trials while minimally blurring the temporal structure, and to reduce the effect of single-trial difficulty. Finally, we applied the standard scaler to normalise the data. Within one species, each session was preprocessed individually and concatenated for further analysis.

### Metrics clustering

We examined the similarity structure between behavioural metrics by computing pairwise Pearson correlation matrices (Hit Rate, Bias, Precision, RT, Speed) separately for each species and on the combined dataset. To visualise metric groupings, we performed hierarchical clustering using 1 - r as a distance measure with Ward’s linkage and plotted the resulting dendrograms alongside the correlation heatmaps. To quantify the separation between speed and accuracy metrics, we cut the dendrogram at two clusters and computed silhouette scores on the correlation-based distance matrix. Across all species and the combined dataset, the five metrics consistently split into an accuracy cluster (Hit Rate, Bias, Precision) and a speed cluster (RT, Speed), with silhouette scores of 0.32 (human), 0.51 (monkey), 0.23 (mouse), and 0.40 (combined).

### HMMs

First, the five metrics were correlated with each other. Based on the resulting correlation analysis, they were divided into two axes: accuracy (hit rate, precision and bias) and speed (reaction time and speed). These two axes provided the input to two hidden Markov models (HMMs). For this analysis, we used an HMM with a Gaussian observation class, implemented in an SSM package [54]. As this is a hypothesis-driven analysis, we chose to split the data into 2 states. The models were trained on all the sessions concatenated, separately for each species. In order to offset state probabilities around the end of one session and the beginning of the next, we added 50 trial padding with a single value across metrics. The padding was reliably classified as a third state and discarded afterwards. Each model classified the trials as belonging to either of two states (accurate/inaccurate for the accuracy model, and slow / fast for the speed model). The conjunction of these classifications resulted in four states, occupying the quadrants of the speed/accuracy space: distracted (slow and inaccurate), deliberate (slow and accurate), efficient (fast and accurate), and impulsive (fast and inaccurate). Thus, each trial was assigned to one of these four states.

### Shuffling

We used shuffling to assess the significance of observed behavioural patterns. The first two preprocessing steps were implemented before the shuffling (see Methods - Data Preprocessing). To create a null distribution, we randomly shuffled the behavioural values of the trials a thousand times. The shuffled trials belonged to the same recording session. We then applied the same data transformations that we used on the original data set, including detrending, smoothing and scaling. Following that, we trained HMMs on each shuffled dataset to create a null distribution for statistical comparisons.

### Cross-species similarity of state fingerprints

For each session, we computed a 20-dimensional “fingerprint” by averaging the five behavioural metrics (hit rate, bias, precision, reaction time, and speed) within each of the four HMM states and concatenating these means. Session fingerprints were then averaged per species. For each species pair we quantified similarity using the Pearson correlation and Euclidean distance between fingerprints, and assessed whether distances were larger than expected by chance using a permutation test that shuffled species labels across sessions (5000 permutations).

### State occupancy across species

For each session and state, we computed state occupancy as the proportion of trials assigned to that state. To test for species differences in occupancy, we used permutation tests on session-level values: a global test per state using the maximum pairwise difference in species means as test statistic, and pairwise tests per state and species pair, obtained by shuffling species labels between the two groups (5000 permutations, two-sided p-values).

### Analysis of dwell times and transitions

Blocks of consecutive trials assigned to the same state were identified, and the times that such blocks lasted were termed the dwell times. Dwell times were statistically compared between the species using a Kruskal-Wallis test, followed by post-hoc Dunn’s tests with Bonferroni correction for multiple comparisons when significant differences were detected. Subsequently, we examined the transitions between the four states. We computed the probabilities of transitioning from each state to any other state, summarised in a transition matrix for each species. As our model features frequent self-transitions (staying in the same state for several trials), we subtracted the diagonal elements from the transition matrices and re-normalised the remaining probabilities. This transformation allowed us to better visualise and analyse the patterns of transitions between different attentional states. To assess whether these transition patterns differed significantly from chance, we performed a permutation test by independently block-shuffling the two constituent state sequences (speed and accuracy) 2,000 times each, and then recombining them into composite states. Block-shuffling preserved the original same-state run durations by permuting only the order of blocks rather than individual trials. For each transition, we computed two-sided p-values by comparing the observed transition probabilities to the null distribution generated by the shuffled sequences, applying FDR (Benjamini-Hochberg) correction for multiple comparisons across off-diagonal cells. To compare transition probabilities across species, we used a per-session species-label permutation test. For a global difference, we computed the Frobenius distance between each pair of species’ transition matrices and compared it to a null distribution generated by permuting session-level species labels (2,000 permutations; all pairs differed globally, p ≤ 0.0015). Specific state-transition differences were assessed per cell by comparing the observed pairwise difference to the permuted null distribution (FDR corrected), with 95% confidence intervals obtained via bootstrap (2,000 resamples).

### Frequency analysis

To identify regular fluctuation patterns in the sequence of behavioural states, we used the time-frequency “superlets” method [23]. Superlets are an extension of the continuous wavelet transform: instead of using a single wavelet at each frequency, they use a set of Morlet wavelets with the same centre frequency but different numbers of cycles. For each time-frequency point, the responses of these wavelets are combined using a geometric mean. This allows for preserving the temporal precision of the shorter wavelets while gaining the better frequency resolution of longer wavelets, achieving “super-resolution” in both time and frequency. This makes it easier to detect short bursts of oscillations compared to standard wavelet or Fourier analysis.

To identify prominent periodic patterns in behavioural data, we focused on states of speed and states of accuracy separately. This way, we could decompose the binary signal (states 1 and 0) into the constituent frequencies. The state labels of all the sessions from a single species were concatenated and then padded with a mirror-reflected signal. In the case of our data, the time unit is one trial. We set the sampling rate to 1000 for convenience. We analysed the frequencies in the range of 6.7 (cycles/1000 trials) to 66.7 (cycles/1000 trials). This is equal to a range of 150–15 trials per cycle. The outcome of this is the frequency power over time for each of the sessions. We then computed an average power value per frequency, averaged across sessions. This resulted in a power spectrum with several peaks.

To assess peak significance, we generated a null distribution from 1000 shuffled datasets (see Methods - Shuffling). We then applied ‘superlets’ to each of the shuffled datasets and computed their power spectra. Values in the real power spectrum falling outside the 2.5th and 97.5th percentiles of this null distribution were considered significant.

To assess differences in the peaks between species, we identified the most prominent peak in the data, which was in the period range of 40–80 trials, and extracted the dominant peak from each session within this range. We then shuffled the species labels 5000 times, subsampling session counts to match each species, and compared the means of real peak distributions to the shuffled means.

## Supporting information

S1 Text Supplementary methods. Details of the reaction time (RT) computation in the VR task, including the sliding-window linear regression with time decay and peak detection procedure used to define RT from lateral movement trajectories.
(PDF)

S1 Fig Performance parameters for each species. Distributions of the five performance parameters are shown for humans (top), monkeys (middle) and mice (bottom).
(TIF)

S2 Fig Correlation matrices and clusters of behavioural parameters. **A)** Correlation matrices between all parameters for humans, monkeys, mice and the combined dataset (left to right). **B)** Hierarchical clustering (Ward’s method, 1 - r distance) of the same correlation matrices shows separate clusters for speed and accuracy parameters in all species, with precision forming its own branch that is closer to the accuracy than to the speed cluster. Silhouette scores indicate moderate cluster separation (human: 0.32, monkey: 0.51, mouse: 0.23, combined: 0.40).
(TIF)

S3 Fig Effects of smoothing. Increasing the window size linearly increased the dwell times for all states and in all species; thus, window size has no effect on the relative dwell times between the states.
(TIF)

S4 Fig Cross-species model. The conjunction of two HMMs was fit on the concatenated, normalised data from all three species. In each panel, the left column shows all trials from the combined dataset, and the remaining columns show only the trials from humans, monkeys, and mice (left to right) assigned by this same cross-species model. **A)** Hit rate versus inverse reaction time, coloured by state. **B)** Mean of each behavioural variable for the four conjunctive states. **C)** Proportion of time spent in each state. **D)** Dwell times per state. Overall, the joint model produces a very similar state structure as the species-specific models, with some minor differences, e.g., mice contributing relatively more trials to the inaccurate states.
(TIF)

S5 Fig 4-state single HMM. Fitting a single 4-state HMM to all five variables (instead of two conjuncted 2-state HMMs) led to state profiles that were hard to interpret. Across the species, the accuracy variables (especially hit rate and bias) dominated the separation between most states, while the speed variables (RT and running speed) varied in a less consistent way across states (top). As a result, the four states did not partition the data cleanly along the hit-rate-RT axes (bottom).
(TIF)

S6 Fig Comparison of individual parameters to HMM states. Plotting all variables against each other and colouring the data by the four states confirms a correct partitioning of the data by the HMM conjunction, for humans (top), monkeys (middle), and mice (bottom).
(TIF)

S7 FigComposite-state transition structure and speed-accuracy switch coupling across species.**A)** Transition matrices including self-transitions for humans (left), monkeys (middle), and mice (right). Diagonal elements (self-transition probabilities around 0.8) are consistent with dwell times of several trials (Fig 4A). **B)** Same matrices after removing self-transitions and renormalizing. Asterisks indicate significant deviations from a null done by independently block-shuffling the speed and accuracy state sequences (BH-FDR ≤ 0.05). **C)** Lagged switch coupling between speed and accuracy sequences. For each species, we computed C(ℓ)=P(accuracy switch at t+ℓ∣speed switch at t) and compared to a circular-shift null. Shaded bands show the 95% null interval; red markers indicate significant lags (BH-FDR corrected).(TIF)

S8 Fig Spectrum in seconds. The power spectra of the state sequence, converted into continuous time by repeating each trial’s state label according to the trial’s duration. Speed states in the top panel, accuracy states in the lower panel. The black line is the average across sessions (dark grey shaded SEM). The dashed grey line and light grey band show the mean and 95% interval of the shuffled control. The vertical dashed line marks the putative location of the largest peaks from the trial-based spectra (55 trials for humans and monkeys, 75 trials for mice, estimated using each species’ median trial duration). We observed power elevations around these locations, even though the amplitude did not exceed the shuffled mean (apart from mice).
(TIF)

S9 Fig Effect of task difficulty on accuracy state transitions. A difficult trial will cause a transition from correct to incorrect in monkeys and humans. The reverse effect is also present.
(TIF)

S10 Fig Relationship between HMM input variables and pupil size. **A)** Almost all of the five HMM variables showed a linear relationship or an inverted U-shape as a function of pupil size, for monkeys (top) and mice (bottom) (human pupil measurements were not taken). **B)** Combining the two speed and three accuracy variables, and plotting the pupil size in the space of those two axes, showed that the pupil was largest when accuracy was low (honeycomb figures; left for monkey, right for mouse). This was confirmed by plotting the average pupil size for the four states (bar plots, left for monkey, right for mouse; note the similarity between the species).
(TIF)

S11 Fig Trial durations. Trial durations in seconds, for the three species. Trial durations are similar between humans and monkeys, and longer in mice.
(TIF)
