# Supplementary material for: Sharing the spotlight: Uncovering common attentional dynamics across species
Source: PLoS Comput Biol. 2026 Apr 22;22(4):e1014191. doi: 10.1371/journal.pcbi.1014191 (PMC13152210; doi:10.1371/journal.pcbi.1014191)
Supplement: S1 Text — Details of the reaction time (RT) computation in the VR task, including the sliding-window linear regression with time decay and peak detection procedure used to define RT from lateral movement trajectories. (PDF) [file pcbi.1014191.s001.pdf]

S1 Text. Supplementary methods

Reaction time

In our VR setting, where the perceptual decision task entails moving towards one of two stimuli, reaction times (RTs) are defined as the time point of the initial substantial movement directed towards either stimulus, irrespective of minor positional adjustments. To calculate the RT, we use a sliding window linear regression approach, incorporating a time decay mechanism. This approach has been described in detail elsewhere [37] and will only be repeated briefly here.

First, we computed a linear regression on the time series of lateral VR movement for adjacent sliding windows, obtaining a measure of local linearity (via R^2 scores) for each window. The resulting array was then analysed for local minima, which are the points where the goodness-of-fit drops beyond a predefined prominence threshold. They serve as lateral movement peaks and are used as reaction time point candidates. For the sake of stability, we used multiple window sizes (85, 160, and 250 ms) to detect these lateral movement peaks and combined the results. These combined results were then transformed into a continuous weight profile by applying a normalised (Gaussian) signal around the time points of each lateral movement peak. Then, we incorporated the time decay by multiplying the continuous array with a linearly decreasing weight vector that biases the analysis toward earlier time points. The time of the largest movement peak in the continuous weight array was then taken as the RT.
